# Samchulkunbi-Tang Alleviates Vascular Endothelial Disorder and Renal Dysfunction in Nitric Oxide-Deficient Hypertensive Rats

**DOI:** 10.1155/2021/8443952

**Published:** 2021-12-17

**Authors:** Mi Hyeon Hong, Jin Seok Hwang, Byung Hyuk Han, Yun Jung Lee, Jung Joo Yoon, Chang Seob Seo, Dae Gill Kang, Hye Yoom Kim, Ho Sub Lee

**Affiliations:** ^1^College of Oriental Medicine and Professional Graduate School of Oriental Medicine, Wonkwang University, 54538 Iksan, Republic of Korea; ^2^Hanbang Cardio-Renal Syndrome Research Center, Wonkwang University, 54538 Iksan, Republic of Korea; ^3^KM Science Research Division, Korea Institute of Oriental Medicine, 34054 Daejeon, Republic of Korea

## Abstract

Samchulkunbi-tang (SCT, Shen Zhu Jian pi tang in Chinese) is said to have been first recorded by Zheng Zhi Zhun Sheng during the Ming Dynasty in China. Records of SCT in Korea are known to have been cited in Donguibogam (Dong Yi Bao Jian in Chinese), Uibang Hwaltu (Yi Fang Huo Tao in Chinese), and Bang Yak Hapyeon (Fang Yao He Bian in China). Although SCT is widely used in treating chronic gastritis and gastric ulcers, the beneficial effect on renal vascular function is unknown. Hypertension is a risk factor for cardiovascular disease and endothelial dysfunction in humans and experimental animal models of arterial hypertension. In addition, kidney dysfunction is characterized by hypertension diseases. This study was conducted to evaluate the effect of SCT on the vascular function *in vitro* (human umbilical cord endothelial cells, HUVECs) and *in vivo* (N^G^‐nitro‐L‐arginine methyl ester, L-NAME-induced hypertensive rats). The phosphorylation of protein kinase B (Akt) and endothelial nitric oxide synthase (eNOS) is closely related to nitric oxide (NO) production in HUVECs, and SCT in this study significantly increased these. For three weeks, hypertensive rat models were induced by L-NAME administration (40 mg/kg/day) with portable water. It was followed by oral administration with 100 and 200 mg/kg/day for two weeks to confirm the effectiveness of SCT. As a result, systolic blood pressure decreased in the SCT-treated groups, compared with that in the L-NAME-induced hypertensive group. SCT treatment restored vasorelaxation by stimulating acetylcholine and cGMP production in the thoracic aorta. In addition, SCT treatment decreased intima-media thickness, attenuated the reduction of eNOS expression, and increased endothelin-1 expression. It also increased p-Akt and p-eNOS expression in hypertensive rat aorta. Furthermore, regarding renal function parameters, SCT ameliorated urine osmolality, urine albumin level, serum creatinine, and blood urea nitrogen levels. These results demonstrate that the oriental medicine SCT exerts potent vascular and renal protective effects on nitric oxide-deficient hypertensive rats and HUVECs

## 1. Introduction

Hypertension, a representative disease of the cardiovascular system, is known to cause conditions such as cardiac hypertrophy and renal dysfunction, thereby reducing cardiovascular function [1]. Damaged blood vessels in hypertension lead to cardiovascular diseases such as heart failure, stroke, and myocardial infarction [2]. Therefore, various studies are being conducted to reduce hypertension's prevalence and increased risk [3]. In hypertensive patients, it is known that there is a correlation with body fluids, which causes heart failure and kidney failure to coexist [4, 5]. Hypertension-induced renal dysfunction is characterized by persistent systemic blood pressure overload, which gradually exceedsIn hypertensive patients, it is known that there is a correlation with body fluids, which causes heart failure and kidney failure to coexist the autoregulatory mechanism to maintain an adequate glute filtration pressure [6]. Therefore, glomerular hypertrophy and proteinuria induced by renal dysfunction are associated with persistent inflammation and cause circulatory dysfunction [7], which leads to endothelial dysfunction [8].

Nitric oxide (NO) is synthesized in endothelial cells and is involved in vasodilation and platelet aggregation prevention. Therefore, it is used to supplement the treatment used for hypertension [9]. The conversion of NO-arginine to citrulline produces NO catalyzed by NO synthase (NOS). It promotes the action of soluble guanylyl cyclase (sGC), which promotes the conversion of guanosine 5′-triphosphate (GTP) to cyclic guanosine 3′,5′-monophosphate (cGMP), and cGMP produced by NO causes smooth muscle relaxation. Endothelial nitric oxide synthase (eNOS) regulates vascular tension and smooth muscle relaxation. Therefore, reduced activity of eNOS can cause right ventricular hypertrophy, hypertension, and arteriosclerosis [10]. N^G^-nitro-L-arginine methyl ester (L-NAME, inhibits NO synthesis) activates the renin-angiotensin system. Activated angiotensin II and soluble guanylyl cyclase cause an increase in blood pressure. Consequently, L-NAME causes blood vessel dysfunction and nephrotic blood vessel contraction. Therefore, L-NAME-induced hypertension inhibits the endothelium-derived NO-cGMP pathway, which leads to the blood vessel, kidney, and heart dysfunction [11].

Hypertension drugs currently used in clinical practice are effective, but they have several side effects when used for a long time. Therefore, many studies are being conducted on alternative medicine as a method to reduce these side effects. Traditional herbal medicine is a valuable alternative treatment for many ailments [12]. Oriental medicinal herbs have long been used as drugs in Asia, especially in China and Korea [13], and the efficacy of traditional Chinese medicine is lower than that of medicine, but they are known to have fewer side effects [14], which was demonstrated by [15, 16]. Samchulkunbi-tang (SCT) is a Korean traditional medicine, documented in a ‘Donguibogam' [17] and called Shen Zhu Jian Pi Tang in China. SCT was used for treating chronic gastritis, gastric ulcer, and gastroptosis [18, 19]. It is comprised of 14 herbs (Table 1). Recently, it was reported to have regulatory roles in the immune system of an ovalbumin-induced murine asthma rat model [19]. Ginseng, a component of SCT, has been reported to have cardiovascular benefits and pharmacological effects such as antioxidation and vasorelaxation [20]. Another component of SCT, *Atractylodes lancea* rhizome, is known to enable blood circulation by inhibiting platelet aggregation [21]. In addition, polysaccharides, a physiologically active ingredient contained in *Poria cocos* Wolf, are known to have an active function in atherosclerosis and inhibit vascular smooth muscle cell proliferation [22]. Many researchers showed that the *magnolia officinalis* cortex has a therapeutic effect on the digestive and cardiovascular systems [23]. *Crataegi Fructus* increases Ginseng radix's bioactive function, and the mixture has the clinical effect of preventing cardiovascular disease [24]. Many studies reported that major components of SCT have the potential to treat cardiovascular function and were related to blood vessels. However, the therapeutic effects of SCT in vascular dysfunction of hypertension have not been demonstrated. Therefore, this study investigates the blood pressure control effect, vascular function, and renal function of SCT in NO deficient hypertensive rats.

## 2. Materials and Methods

### 2.1. Reagents and Drug Administration

Samchulkunbi-tang (SCT) consisted of 14 medicinal herbs provided by the Korean Institute of Oriental Medicine (Table 1). L-NAME, acetylcholine (ACh), phenylephrine (PE), and sodium nitroprusside (SNP) were purchased from Sigma (Sigma Chemical Co., St. Louis, MO, USA). Olmesartan medoxomil (Olmetec®) was purchased from Daewoong Pharm. Co. (Seoul, Korea). The endothelial nitric oxide synthase (eNOS, NOS3) antibody was from Invitrogen Cellular Analysis (Carlsbad, CA, USA). Phospho-eNOS, protein kinase B (Akt), phosphor-Akt, *β*-actin, and endothelin-1 (ET-1) antibodies were purchased from Santa Cruz Biotech (Santa Cruz Biotechnology, Inc., Dallas, TX, USA).

### 2.2. High-Performance Liquid Chromatography (HPLC) Analysis of SCT

The HPLC analysis of SCT was attended utilizing the HPLC system and LC Solution software (Ver. 1.24 SP1, Shimadzu, Kyoto, Japan). Phenomenex Gemini column (C_18,_ 250 × 4.6 mm; 5 *μ*m, Torrance, CA, USA) was used to separate the seven marker components while held at 40°C. The mobile phases for the efficient separation of the seven marker compounds consisted of solvent A (distilled water) and solvent B (acetonitrile), containing 1.0% acetic acid, with gradient elution. Analysis was performed at an injection volume of 10 ml and a 1.0 mL/min flow rate.

### 2.3. Cell Cultures and Intracellular Nitric Oxide Production

Human umbilical vein endothelial cells (HUVEC) were cultured in media (Endothelial Cell Basal Medium-2, Promo Cell, Heidelberg, Germany) at a density of 5 × 10^5^ cells/ml. To detect intracellular NO production, we performed the measurements using the NO-sensitive fluorescent dye DAF-2DA (Merck Biosciences, Schwalbach, Germany). HUVECs grown in 6 well plates were treated with SCT for 30 minutes. After that, DAF was added for the final 30 min (incubation). Reactions were stopped by fixing the cells in 2% of paraformaldehyde for 30 min (at RT). Fluorescence intensity was measured using an Eclipse Ti (Eclipse Ti fluorescence microscope, Nikon Corporation, Tokyo, Japan).

### 2.4. L-NAME-Induced Hypertension Rat Experimental Animal Preparation

Animal procedures were performed by the National Institute of Health Guidelines for the care and use of laboratory animals. Animal procedures were approved by the Animal Care and Utilization Committee of the Animal Institution of Wonkwang University (WKU17-60). In previous studies, we conducted a study on the antihypertensive effect derived from natural products that can minimize side effects based on herbal medicines known in the old literature [25]. A total of 50 herbal medicines were tested for vascular relaxation (ex vivo), and one of the effective drugs was Samchulkunbi-tang. Therefore, based on the vasorelaxation effect shown in the results of this study, it was thought that it would be worth conducting an in-depth study, so an in vivo experiment was conducted. Male Sprague Dawley rats (150–220 g) were purchased from KOATECH (Pyeongtaek, Korea). After 7 days of adaptation, L-NAME was added to drinking water and administered for 5 weeks, and the control group was excluded. The rats were divided into 5 experimental groups: CONT, vehicle; L-NAME (40 mg/kg/day); OMT, L-NAME + olmetec (10 mg/kg/day); SCTL, L-NAME + SCT (100 mg/kg/day); SCTH, L-NAME + SCT (200 mg/kg/day). OMT and SCT were orally administered daily for 3 weeks.

### 2.5. Measurement of Blood Pressure Changes and Isometric Vascular Tone Changes

Systolic blood pressure (SBP) was assessed once a week for 5 weeks by tail-cuff blood pressure monitor (MK2000; Muromachi Kikai, Tokyo, Japan) under a quiet and warm room. The SBP data was measured at least 10 times to determine the average value. In this study, SBP higher than 160 mmHg was used. The aortic ring isolated from the rat was hung using a triangular shape stainless steel wire in a chamber containing 5 mL Krebs solution (pH 7.4). Changes in the tension of the aortic ring were recorded via a Grass transducer (Grass Force Transducer FT03, Grass Instrument Company, Quincy, MA, USA). Acetylcholine (ACh) and sodium nitroprusside (SNP) were treated by concentration to examine relaxation changes between each group.

### 2.6. Measurement of Western Blot Analysis and cGMP Levels

After separating the aortic tissue in rats, the tissue was frozen in liquid nitrogen (LN_2_) and stored at −70°C. Protein was separated from thoracic aorta tissue or cell, and cell homogenates were loaded onto SDS-polyacrylamide gel (8–12%) electrophoresis and transferred to nitrocellulose membranes paper. The membranes paper was blocked with BSA powder containing TBS for 2 hours and incubated overnight at 4°C with primary antibody. After incubating the appropriate secondary antibody for 1 hour, the membrane bands were visualized (ECL solution, Amersham, Buckinghamshire, UK). Protein band images were taken and analyzed using the Invitrogen iBright Imaging systems (Thermo Fisher Scientific, Waltham, MA, USA). In addition, the thoracic aorta was homogenized with 0.1 MHCl, and then the cGMP level was measured using the supernatant obtained by centrifugation. According to the manufacturer's instructions, thoracic aorta cGMP levels were determined using the cGMP ELISA kit (Enzo, ADI-900-014, Farmingdale, NY, USA).

### 2.7. Histopathological Investigations

After fixing the thoracic aortic tissue with 10% (v/v) formalin, it was dehydrated and embedded in paraffin. The thoracic aortic tissue of each group was sliced into four *μ*M thick and attached to the slide (Thermo Fisher Scientific, Pittsburgh, PA, USA). Hematoxylin and eosin (H&E) stain and immune stains (Histostain® Plus Broad Spectrum Kit, Thermo Fisher Scientific, Waltham, MA, USA) were performed for pathological tissue comparison. The expressions of eNOS (Santa Cruz, CA, USA) and ET-1 (Santa Cruz, CA, USA) were identified in the thoracic aortic section. Image J (NIH, Bethesda, MD, USA), NIH Image analysis software, was used to calculate the quantity of protein expression and aortic wall thickness.

### 2.8. Investigation of Renal Function Parameters

Experimental animals were kept in metabolic cages for 24 hours to measure water intake and collect urine (Rodent Metabolic Cage System, TECNIPLAST, Buguggiate, Italy). Urine samples were collected to measure urine osmolality (Osmometer, Advanced Instruments, Norwood, MS) and urine albumin levels. In addition, after the experiment was completed, a blood sample was collected in a test tube containing ethylene-diamine-tetraacetic acid (EDTA) and centrifuged at 4°C for 15 minutes (stored at −70°C). The creatinine, albumin, and blood urea nitrogen (BUN) levels in plasma were measured using the kit (ARKRAY, Inc., Kyoto, Japan).

### 2.9. Statistical Analysis

Significant differences were compared using the repeated measures ANOVA multiple comparison test. Student's *t*-test on paired or unpaired data was also applied. All statistical analyses were conducted using Sigma Plot 10 (Systat Software, Inc., Chicago, IL, USA). Statistical significances between groups were defined as *p* < 0.05. All results were given as mean ± standard error (S.E.).

## 3. Results

### 3.1. HPLC Analysis Result of Samchulkunbi-Tang (SCT)

**Figure 1:**
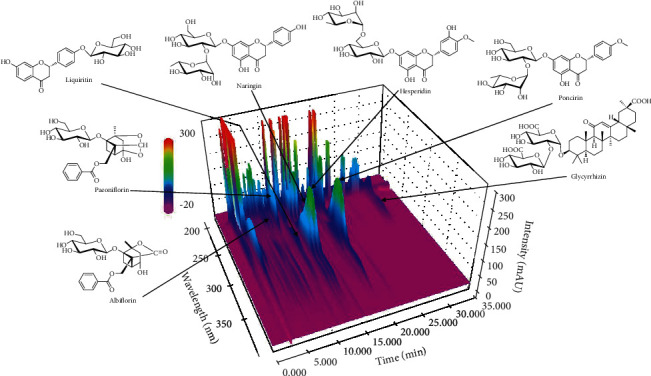
HPLC analysis of Samchulkunbi-tang (SCT) sample.

Optimized HPLC-PDA methods were used to identify and quantify seven marker compounds in the SCT sample. Each marker was confirmed by comparing the UV spectrum and retention time to a reference standard. Paeoniflorin, naringin, albiflorin, liquiritin, hesperidin, glycyrrhizin, and poncirin were determined to account for about 8.77, 9.66, 11.49, 13.47, 13.98, 19.13, and 30.30%, respectively, of the SCT sample (Figure 1). The concentrations of liquiritin, albiflorin, paeoniflorin, hesperidin, naringin, glycyrrhizin, and poncirin were 1.39, 5.55, 1.61, 4.98, 6.76, 5.44, and 2.78 mg/freeze-dried g, respectively.

### 3.2. Effect of SCT on Nitric Oxide Production in Human Umbilical Vein Endothelial Cells

**Figure 2:**
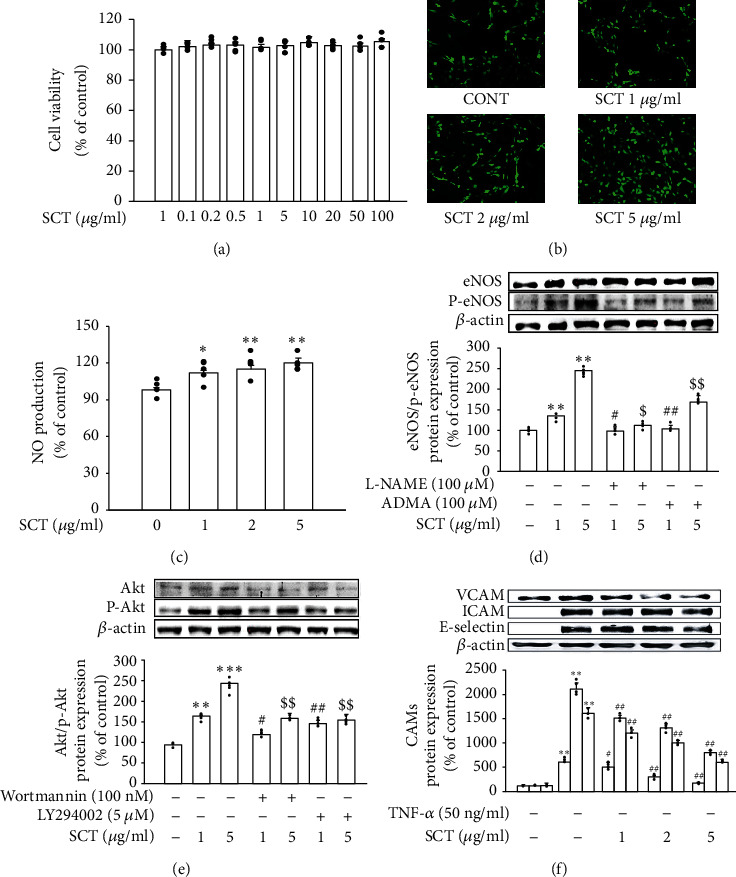
Effect of SCT on NO production, phosphorylated eNOS, and Akt in HUVEC. (a) Effect of SCT on the viability of cells (HUVEC). SCT (0–100 *μ*g/mL) was treated for 24 hours. (b) NO production was investigated through a fluorescence microscope (magnification, ×100). (c) The nitric oxide production of each group was analyzed as a Griess reaction. (d) Effect of SCT on p-eNOS expression was confirmed by western blot. (e) Effect of SCT on p-Akt expression was analyzed by western blot. (f) Effect of SCT on the expression of vascular inflammatory factors in HUVECs. SCT was pretreated for 30 minutes and then stimulated with TNF-*α* (50 ng/mL). Protein expression of inflammatory factors (ICAM-1, VCAM-1, and E-selectin) was confirmed using Western blotting. Values are expressed as mean ± S.E. Student's *t*-test on paired or unpaired data was also applied. All statistical analyses were conducted using Sigma Plot 10. ^∗∗^*p* < 0.01 vs. CONT, ^##^*p* < 0.05 vs. SCT 1 *μ*g/mL, and ^$$^*p* < 0.05 vs. SCT 5 *μ*g/mL.

In this study, SCT (0–100 *μ*g/ml) was treated for 24 hours to determine the effect of SCT on cell viability (HUVEC), and no toxicity was shown (Figure 2(a)). In addition, as a result of measuring the level of nitric oxide (NO) produced in HUVECs after treatment with SCT, it was confirmed that NO production increased in a dose-dependent manner in SCT (Figures 2(b) and 2(c)). After 30 minutes of pretreatment of different doses of SCT in endothelial cells, the phosphorylation of eNOS and Akt was confirmed by Western blot analysis. In addition, L-NAME (N^G^-nitro-L-arginine methyl ester) and asymmetric dimethylarginine (ADMA) were treated as NOS3 inhibitors, and LY294002 and wortmannin were treated as Akt inhibitors. The phosphorylated eNOS and Akt expression increased in SCT dose-dependently, suggesting that SCT stimulates p-eNOS and p-Akt expression in HUVECs (Figures 2(d) and 2(e)). Furthermore, SCT was pretreated for 30 minutes and then stimulated with TNF-*α* (50 ng/mL). In addition, after pretreatment with SCT for 30 minutes, the protein expression of inflammatory factors (ICAM-1, VCAM-1, and E-selectin) was confirmed through stimulation with TNF-*α* (50 ng/ml). SCT has been shown to reduce the expression of vascular inflammatory factors in HUVECs (Figure 2(f)).

### 3.3. Effect of SCT on Decreased Blood Pressure and Attenuated Blood Vessel Function

**Figure 3:**
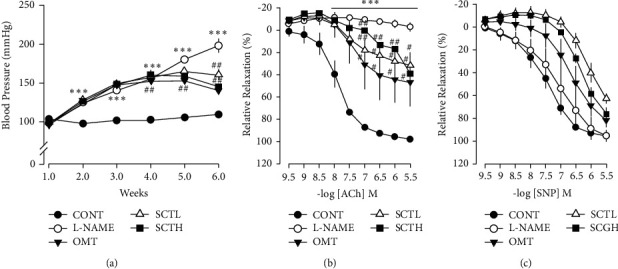
Effect of aortic dysfunction improvement SCT in L-NAME hypertensive rats. (a) Systolic blood pressure measured by tail-cuff method (mmHg). Values are expressed as mean ± S.E. (*n* = 8 per group). (b) Endothelium-dependent relaxation to Ach in thoracic aorta of each group. (c) Endothelium-dependent relaxation to SNP in thoracic aorta of each group. Values are expressed as mean ± S.E. Student's *t*-test on paired or unpaired data was also applied. All statistical analyses were conducted using Sigma Plot 10. ^*∗∗*^*p* < 0.01, ^*∗∗∗*^*p* < 0.001 vs. CONT; ^#^*p* < 0.05, ^##^*p* < 0.01 vs. L-NAME.

As a result of evaluating the effect of SCT on blood pressure in hypertensive rats, L-NAME-induced hypertensive rats increased steadily for five weeks compared to control rats (Figure 3(a)). From 3 weeks, olmetec (10 mg/kg/daily) and SCT (100 and 200 mg/kg/daily) were orally administered to the L-NAME-induced hypertensive rats. As a result of confirming the vasodilatory response to ACh by isolating the thoracic aorta, the vasodilatory response was significantly reduced in the L-NAME group compared to the control group and normalized in the SCT-treated group (Figure 3(b)). On the other hand, the vasodilatory response by SNP did not have a significant effect between all groups (Figure 3(c)).

### 3.4. SCT Alleviated eNOS, Akt1/2/3 Protein Expression, and cGMP Production

**Figure 4:**
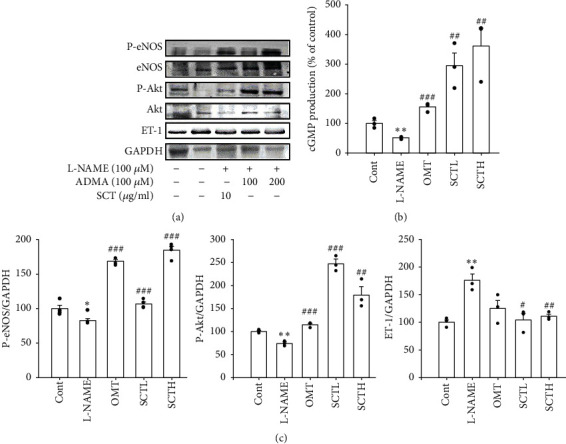
Effect of SCT on the expression of Akt, eNOS, ET-1, GAPDH, and cGMP production in aorta of L-NAME hypertensive rats. (a) SCT has influence on expression of eNOS and phosphorylation of eNOS, phosphorylated Akt1/2/3, and ET-1. (b) cGMP production in thoracic aorta of L-NAME hypertensive rats was measured by ELISA kit. Values are expressed as mean ± S.E. Student's *t*-test on paired or unpaired data was also applied. All statistical analyses were conducted using Sigma Plot 10. ^*∗*^*p* < 0.05, ^*∗∗*^*p* < 0.01 vs. CONT; ^#^*p* < 0.05, ^##^*p* < 0.01, and ^###^*p* < 0.001 vs. L-NAME.

Phosphorylated eNOS and Akt1/2/3 expression were significantly reduced in the L-NAME group compared with the control group, and eNOS expression in the SCT (200 mg/kg/day) treatment group was significantly restored to the control group level (Figure 4(a)). ET-1 expression was increased in the L-NAME group compared to the control and significantly decreased with treatment of SCT (Figure 4(a)). In addition, the decreased cGMP levels in the L-NAME hypertensive group compared to the control group were significantly increased in the SCT-treated group (Figure 4(b)).

### 3.5. SCT Has Changed Thoracic Aorta Morphology, eNOS, and ET-1 Expression

**Figure 5:**
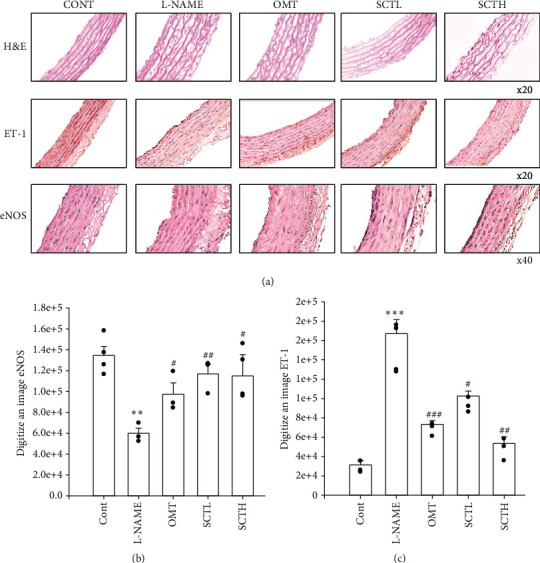
Effect of SCT on aorta morphology and eNOS and ET-1 immunoreactivity in aortic tissues. Hematoxylin and eosin were represented, and immunoreactivity of eNOS and ET-1 expression was shown by microscope. Values are expressed as mean ± S.E. Student's *t*-test on paired or unpaired data was also applied. All statistical analyses were conducted using Sigma Plot 10. ^*∗∗*^*p* < 0.01 vs. CONT; ^#^*p* < 0.05, ^##^*p* < 0.01, and ^###^*p* < 0.001 vs. L-NAME (magnification ×200 or ×400).

The intimal endothelial and medial layers of rats of the L-NAME group increased compared with those of the control group rats. In the SCT-treated groups, especially the 200 mg/kg/day (high concentration) group, the intimal and medial layers of the thoracic aorta decreased, compared with those in the L-NAME group (Figure 5(a)). The expression of eNOS in the thoracic aorta was decreased in the L-NAME hypertensive group compared to the control group but was alleviated in the SCT-treated group (Figures 5(a) and 5(b)). The expression of ET-1 in the thoracic aorta was increased in the L-NAME-induced hypertension group compared to the control group but decreased significantly in the SCT-treated group (Figures 5(a) and 5(c)).

### 3.6. SCT Alleviated Urinary and Renal Function Parameter

In order to confirm the effect of SCT treatment on kidney function, rats of all groups were placed in a metabolic cage to collect water intake and urine. As a result, water intake increased in the L-NAME group compared to the control group but decreased in the SCT-treated group. As a result, the urine excretion amount of rats in the L-NAME group increased compared to the control group, and the SCT group significantly decreased compared to the L-NAME group (Table 2). The urine collected from metabolic cages was analyzed to determine osmolality, albumin, and BUN. As a result, the osmolality of the L-NAME group was decreased compared to the control group, and it was found that there was a significant improvement effect in the SCT treatment group (Table 2). In addition, it was confirmed that the increased albumin concentration in L-NAME hypertensive group was normalized in SCT-treated group (Table 2). Plasma albumin, BUN, and creatinine concentration were significantly decreased in the L-NAME group compared to the control group, and albumin concentration was recovered in the SCT-treated group (Table 3). Therefore, SCT treatment is thought to improve renal function in L-NAME-induced hypertensive rats.

## 4. Discussion

Samchulkunbi-tang (SCT) was initially used to treat indigestion and regulate the immune system in Korean medicine. However, the protective effect of SCT on blood circulation disorders, including hypertension, is unknown. This study is the first to elucidate that SCT has beneficial effects against vascular and renal dysfunction in nitric oxide (NO)-deficient hypertensive rats.

It is known that inhibition of NO production by L-NAME treatment causes hypertension [26]. Therefore, our study was conducted using L-NAME-induced hypertensive rat model with reference to previous studies [26, 27]. This treatment impaired NO signaling, which in turn decreased NO production in the arteries of the rats. Smooth muscle tension is weakened by the inhibition of NO production, resulting in increased peripheral resistance and peripheral blood vessel contraction [28]. We measured blood pressure every week to determine the degree of hypertension induced by L-NAME. This study demonstrated that it was possible to judge that hypertension was sufficiently induced because systolic blood pressure showed significant increased effect when compared with the control group, thus suggesting that SCT had an antihypertensive effect. Moreover, acetylcholine (ACh) regulates vascular relaxation by activating endothelial nitric oxide (eNOS) synthesis by causing NO release through specific endothelial receptors. In other words, endothelial function regulates vascular relaxation [29, 30]. Acetylcholine-induced vasodilation was significantly inhibited in the L-NAME group compared to the control group but was recovered by SCT treatment. SNP is reported to directly reduce Ca^2+^ concentration and relax smooth muscles [31]. In this study, the SNP-induced relaxation rate differed, suggesting that SCT-induced vasorelaxation results from restored endothelial damage. NO produced in endothelial cells activates guanylate cyclase and promotes vascular relaxation by increasing cGMP production in vascular smooth muscle [32]. In this study, the decreased cGMP level in the L-NAME group was recovered by SCT treatment, and it is believed that the vascular relaxation effect by SCT in the smooth muscle is affected by the increase in cGMP production.

Several studies have reported that L-NAME-induced hypertensive rats exhibited left ventricular hypertrophy, left ventricular fibrosis, and increased thoracic aortic wall thickness [33, 34]. It was observed that the aortic thickness was increased in the L-NAME group compared to the rats in the control group, and it was attenuated by SCT treatment. Increased eNOS activity in blood vessels leads to relaxation of the vessels, implying that endothelial function is closely related to blood pressure [35, 36]. Immunohistochemical staining and western blot analysis suggested that eNOS expression in the thoracic aorta was attenuated in the L-NAME group and was weakly ameliorated in the SCT-treated group. ET-1, an endothelial vasoconstrictor, increased vascular tone [37, 38] and was associated with hypertension, ischemic heart disease, and congestive heart failure [39]. Thus, the expression level of ET-1 was increased in the L-NAME group, and SCT decreased it to improve vascular function. These results suggest that the improvement of the ACh-induced vascular relaxation effect of SCT in the L-NAME hypertension animal model is due to the eNOS/cGMP pathway activation.

Renal dysfunction has been reported to be associated with cardiovascular disease or coronary artery disease [40]. Increased serum BUN level represents renal dysfunction, which could provide supplementary information on hypertension [41]. Reportedly, high volume and low osmolality of urine are risk factors for kidney dysfunction [42]. In addition, chronic blockade of NO synthesis (by L-NAME administration) causes systemic hypertension, renal vasoconstriction, proteinuria, and glomerulosclerosis damage [43]. In addition, serum albumin and creatinine levels are considered indicators of renal dysfunction in hypertension, and it is known that albumin decreases [44, 45], and creatinine increases in renal function abnormalities by hypertension [46]. In the study, BUN levels increased in the L-NAME group but weakened after SCT administration. On the other hand, urine osmolality was decreased in the L-NAME group compared to the control group, but it improved in the SCT-treated group. It is thought that SCT has an improvement effect on renal function. In this study, the abnormality of renal function parameters was improved by SCT treatment, suggesting that SCT can restore renal function in hypertension. Endothelial dysfunction is characterized by decreased NO production and is an essential early mediator linked to obesity and cardiovascular disease [47]. Akt downstream of PI3K is also considered essential for cell survival, and Akt activation in endothelial cells has been reported to promote cell survival [48]. The results of this study confirmed that SCT promotes NO production, thus promoting cell survival. This study also studied the PI3K/Akt pathway and phosphorylation of eNOS in the anti-inflammatory effect of SCT. eNOS and Akt phosphorylation were also increased by SCT. These results provide strong evidence of an anti-inflammatory effect of SCT through the PI3K/Akt-dependent eNOS pathway.

## 5. Conclusion

**Figure 6:**
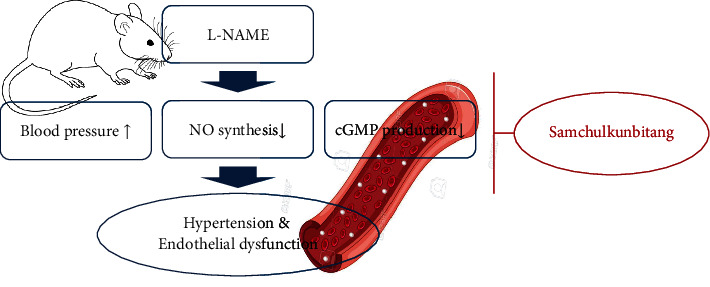
SCT regulates blood pressure through an endothelial-derived pathway and improves renal dysfunction and vascular inflammatory processes in L-NAME-induced hypertensive rats.

These results suggest that SCT regulates blood pressure through an endothelial-derived pathway and improves renal dysfunction and vascular inflammatory processes in L-NAME-induced hypertensive rats. Therefore, it can be seen that SCT improves cardiovascular and renal dysfunction in hypertension (Figure 6).

## Figures and Tables

**Table 1:** Composition of Samchulkunbi-tang (SCT).

Herbal medicine	Scientific name	Family	Purchase	Weight (g)
Ginseng radix	*Panax ginseng* C. A. Meyer	Araliaceae	Omniherb	3.750
Atractylodis rhizoma	*Atractylodes japonica* Koidzumi	Compositae	Omniherb	3.750
Poria sclerotium	*Poria cocos* Wolf	Polyporaceae	Omniherb	3.750
Magnoliae cortex	*Magnolia officinalis* Rehder et Wilson	Magnoliaceae	HMAX	3.750
Citri unshius pericarpium	*Citrus unshiu* Markovich	Rutaceae	Omniherb	3.750
Crataegi fructus	*Crataegus pinnatifida* Bunge	Rosaceae	Omniherb	3.750
Ponciri fructus immaturus	*Poncirus* trifoliata Rafinesque	Rutaceae	HMAX	3.000
Paeoniae radix	*Paeonia lactiflora* Pallas	Paeoniaceae	Omniherb	3.000
Amomi semen	*Amomum villosum* Loureiro	Zingiberaceae	HMAX	1.875
Massa medicata fermentata	—	—	Omniherb	1.875
Hordei fructus germinatus	*Hordeum vulgare* Linne var. *hexastichon Aschers*	Gramineae	Omniherb	1.875
Glycyrrhizae radix et rhizoma	*Glycyrrhiza uralensis* Fischer	Leguminosae	HMAX	1.875
Zingiberis rhizoma recens	*Zingiber officinale* Roscoe	Zingiberaceae	Omniherb	3.750
Zizyphi fructus	*Zizyphus jujube* Miller var. *inermis* Rehder	Rhamnaceae	Omniherb	3.750
**Total amount**				**43.500**

**Table 2:** Effect of SCT on urine osmolality changes in L-NAME-induced hypertensive rats.

	Cont.	L-NAME	OMT 10 (mg/kg/day)	SCT 100 (mg/kg/day)	SCT 200 (mg/kg/day)
Body weight (g)	359.6 ± 5.77	320.7 ± 9.7^*∗∗*^	360.1 ± 4.2^##^	362.5 ± 4.46^#^	360.6 ± 7.9^##^
Water intake	22.4 ± 1.3	35.04 ± 1.8^*∗∗∗*^	25.3 ± 3.9^##^	30.8 ± 6.1^#^	26.4 ± 6.4^#^
Urine volume (g/23 h)	33.2 ± 0.8	45.5 ± 5.2^*∗*^	30.8 ± 1.8^#^	31.18 ± 4.5^#^	35.14 ± 1.9^#^
Osmolality (mOsm/kg H_2_O)	2262.7 ± 110.3	795 ± 79.9^*∗∗∗*^	1932.7 ± 19.5^###^	1399 ± 83.0^##^	1503.7 ± 101.7^##^
Albumin (mg/dl, 23 h)	0.487 ± 0.027	0.615 ± 0.032^*∗*^	0.483 ± 0.037^#^	0.529 ± 0.036	0.451 ± 0.034^##^

SCT, Samchulkunbi-tang; CONT, control; L-NAME, L-NAME-treated 40 mg/kg/day; OMT, L-NAME + OMT 10 mg/kg/day; SCTL, L-NAME + SCT 100 mg/kg/day; SCTH, L-NAME + SCT 200 mg/kg/day. The data of values show mean ± S.E. (*n* = 8 per group). ^*∗∗*^*p* < 0.05, ^*∗∗*^*p* < 0.01, and ^*∗∗∗*^*p* < 0.001 vs. CONT; ^#^*p* < 0.05, ^##^*p* < 0.01, and ^###^*p* < 0.001 vs. L-NAME.

**Table 3:** Effect of SCT on renal function parameter changes in L-NAME-induced hypertension rats.

	Cont	L-NAME	OMT 10 (mg/kg/day)	SCT 100 (mg/kg/day)	SCT 200 (mg/kg/day)
Albumin (g/dl)	3.4 ± 0.05	3.02 ± 0.12^*∗∗*^	3.78 ± 0.1^###^	3.35 ± 0.2^##^	3.6 ± 0.2^##^
BUN (mg/dl)	14.7 ± 0.9	51 ± 13.0^*∗∗∗*^	22.9 ± 4.2^##^	34 ± 11.5^###^	29.5 ± 15.5^##^
Creatinine (mg/dl)	0.7 ± 0.05	1.21 ± 0.2^*∗∗∗*^	1.15 ± 0.14	0.95 ± 0.1^###^	0.9 ± 0.1^###^

SCT, Samchulkunbi-tang; CONT, control; L-NAME, L-NAME-treated 40 mg/kg/day; OMT, L-NAME + OMT 10 mg/kg/day; SCTL, L-NAME + SCT 100 mg/kg/day; SCTH, L-NAME + SCT 200 mg/kg/day. The data of values show mean ± S.E. (*n* = 8 per group). ^*∗∗*^*p* < 0.01 and ^*∗∗∗*^*p* < 0.001 vs. CONT; ^##^*p* < 0.01, and ^###^*p* < 0.001 vs. L-NAME.

## Data Availability

The datasets used and/or analyzed during the current study are available from the corresponding author upon reasonable request.

## Abbreviations

ACh: Acetylcholine
cGMP: Cyclic guanosine 3′, 5′-cyclic monophosphate
BUN: Blood urea nitrogen
DW: Distilled water
eNOS: Endothelial nitric oxide synthase
ET-1: Endothelin-1
H&E: Hematoxylin and eosin
IHC: Immunohistochemistry
L-NAME: N^G^-Nitro-L-arginine methyl ester
NO: Nitric oxide
NOS: Nitric oxide synthase
PE: Phenylephrine
SBP: Systolic blood pressure
SCT: Samchulkunbi-tang
SNP: Sodium nitroprusside.
